# Delay in diagnosis of pulmonary tuberculosis increases the risk of pulmonary cavitation in pastoralist setting of Ethiopia

**DOI:** 10.1186/s12890-019-0971-y

**Published:** 2019-11-06

**Authors:** Fentabil Getnet, Meaza Demissie, Alemayehu Worku, Tesfaye Gobena, Rea Tschopp, Michael Girmachew, Gebeyehu Assefa, Berhanu Seyoum

**Affiliations:** 1 College of Medicine and Health Sciences, Jigjiga University, Jijiga, Ethiopia; 2 Addis Continental Institute of Public Health, Addis Ababa, Ethiopia; 3 School of Public Health, Addis Ababa University, Addis Ababa, Ethiopia; 4 School of Public Health, Haramaya University, Dire Dawa, Ethiopia; 5 Armauer Hansen Research Institute, Addis Ababa, Ethiopia; 6 Swiss Tropical and Public Health Institute, Basel, Switzerland; 7 University of Basel, Basel, Switzerland; 8 Karamara Referral Hospital, Jijiga, Ethiopia

**Keywords:** Diagnosis delay, Tuberculosis, Cavitation, Smear positivity, Pastoralist, Ethiopia

## Abstract

**Background:**

Delay in diagnosis and treatment of pulmonary tuberculosis (PTB) leads to severe disease, adverse outcomes and increased transmission. Assessing the extent of delay and its effect on disease progression in TB affected settings has clinical and programmatic importance. Hence, the aim of this study was to investigate the possible effect of delay on infectiousness (cavitation and smear positivity) of patients at diagnosis in Somali pastoralist area, Ethiopia.

**Methods:**

A cross-sectional study was conducted between December 2017 and October 2018, and 434 newly coming and confirmed PTB patients aged ≥15 years were recruited in five facilities. Data were collected using interview, record-review, anthropometry, Acid-fast bacilli and chest radiography techniques. Log-binomial regression models were used to reveal the association of delay and other factors associated with cavitation and smear positivity, and ROC Curve was used to determine discriminative ability and threshold delays.

**Results:**

Median age of patients was 30 years. Of all, 62.9% were males, and 46.5% were pastoralists. Median diagnosis delay was 49 days (IQR = 33–70). Cavitation was significantly associated with diagnosis delay [*P* < 0.001]; 22.2% among patients diagnosed within 30 days of illness and 51.7% if delay was over 30 days. The threshold delay that optimizes cavitation was 43 days [AUC (95% CI) = 0.67(0.62–0.72)]. Smear positivity was significantly increased in patients delayed over 49 days [*p* = 0.02]. Other factors associated with cavitation were age ≤ 35 years [APR (95% CI) =1.3(1.01–1.6)], chronic diseases [APR (95% CI) = 1.8(1.2–2.6)] and low MUAC*^female^ [APR (95% CI) = 1.8(1.2–2.8)]. Smear positivity was also associated with age ≤ 35 years [APR (95% CI) =1.4(1.1–1.8)], low BMI [APR (95% CI) =1.3(1.01–1.7)] and low MUAC [APR (95% CI) =1.5(1.2–1.9)].

**Conclusion:**

This study highlights delay in diagnosis of pulmonary TB remained high and increased infectiousness of patients in pastoral settings of Ethiopia. Hence, delay should be targeted to improve patient outcomes and reduce transmission in such settings.

## Background

Tuberculosis (TB) remains the leading killer of infectious diseases. Globally, it caused an estimated 10 million cases and 1.3 million deaths in 2017. Ethiopia ranked 11th among the 22 high TB burden countries and 4th in Africa with around 172, 000 new cases in 2017 [1]. The disease is more prevalent in pastoral communities of the country [2]. The End-TB strategy sets early diagnosis and prompt treatment of cases as pillars to ending the global epidemics by 2030 [3]. Particularly in Ethiopia, the national TB control program (NTP) primarily focuses on detection of presumptive TB cases who present themselves to health facilities, *aka passive case finding strategy* [4].

However, this passive approach struggles to achieve the required case detection rates in resource-limited settings, leaving millions of potentially infectious cases undiagnosed in communities [5, 6]. Nearly one-third of TB cases in Ethiopia were not detected in 2017 [1]. The number of undetected cases could be equal or more than the number of cases detected by the healthcare system in certain communities of the country [6, 7]. In Somali Regional State of Ethiopia (SRS), TB case detection rates have never exceeded 50% in the past years [8]. The problem is likely influenced by healthcare seeking behavior of patients or health system deficiencies [9, 10]. Patients may delay without seeking healthcare, or the healthcare providers may fail to identify presented cases [11].

Delay in diagnosis and treatment leads to severe disease, death, and facilitates transmission in households and congregate settings. Fail to timely treat PTB leaves destructive damages to lung tissues, the classical hallmark is pulmonary cavitation [12, 13]. The cavities formed are caves for *Mycobacterium tuberculosis (MTB)* and release higher bacilli load in aerosols [14], which is the channel of transmission. Cavities also slow smear conversion (prolongs contagious period), cause adverse treatment outcomes, and leave permanent lung damages even after successful treatment [15]. Along with disease progression, delay also extends contagious period and contact time between patients and susceptible contacts [16].

This highlights assessment of delay in medical care and its impact is very helpful to evaluate the effectiveness of TB programs in controlling the disease and its transmission in affected communities. There is also limited data on the prevalence of cavitary TB and the tolerable delays from clinical and programmatic perspectives in Ethiopia. Hence, the aim of this study was to evaluate the possible effect of diagnosis delay on pulmonary cavitation and smear positivity as proxy measures of patient infectiousness in Somali Regional State of Ethiopia, a pastoralist predominated area.

## Methods

### Study setting

Four hospitals (Kharamara, Dege-habour, Kebri-Daher and Gode) and one health center (Abilelie) were included based on their high patient flow, presence of radiologic facility, and geographic location in Somali Regional State of Ethiopia. Approximately 85% of the region’s population are rural and lead pastoral life that is characterized by seasonal migration following climatic conditions [17]. The health facilities provide TB diagnosis services as per the national guideline, which involves two spot-spot smear microscopy spaced by 30 min (and morning on demand), chest radiography, GeneXpert, pathology and clinical investigations [4].

### Study design and population

A facility-based cross-sectional study was conducted between December 2017 and October 2018. Newly diagnosed PTB patients aged ≥15 years were included regardless of smear status and treatment category. Patients aged ≥15 years manifest similar pathological features and the same diagnosis approaches are followed [4]. People in this age category acquire competent immunity that is key in cavity formation [18], cover 80% of all TB cases, and account for almost 100% of transmissions [19]. Patients with other pulmonary co-morbidities (bronchitis, pneumonia and lung cyst) were excluded.

### Sample size and sampling technique

The minimum sample size estimated was 282 using OpenEpi303 software for cross-sectional studies. The assumptions were 95% CI, 80% power, 1:1 ratio of non-delayed/delayed, 27.5% of non-delayed and 45% of delayed patients had cavitation in related study [12], 5% precision and 10% non-response rate, given delay above 30 days as critical point at which risk of transmission increases [20]. We included all the available samples in the analysis to increase the power of the test, which raised the final sample size to 434. Newly upcoming PTB patients during the study period were recruited immediately after diagnosis and before treatment initiation.

### Data collection

A mix of methods was used to collect data including face-to-face interview, record review, anthropometry, Acid-Fast Bacilli (AFB) and chest radiography. Interviews were conducted using a structured questionnaire (translated into native Somali and Amharic languages), questions were adapted from multinational study in Eastern Mediterranean WHO region [21] and related studies in Ethiopia [11, 22, 23]. Records were reviewed to substantiate co-morbidities and medical history. Anthropometry measurements were also taken. Upon completion of interviews and anthropometry, patients were linked to laboratory and radiology units using request forms prepared for this purpose. Nurses working in DOTS clinics carried out recruitment, interview, record review and anthropometry procedures. Training was provided on sampling and data collection procedures by the principal investigator and a local research assistant.

### AFB examination

**Fig. 1:**
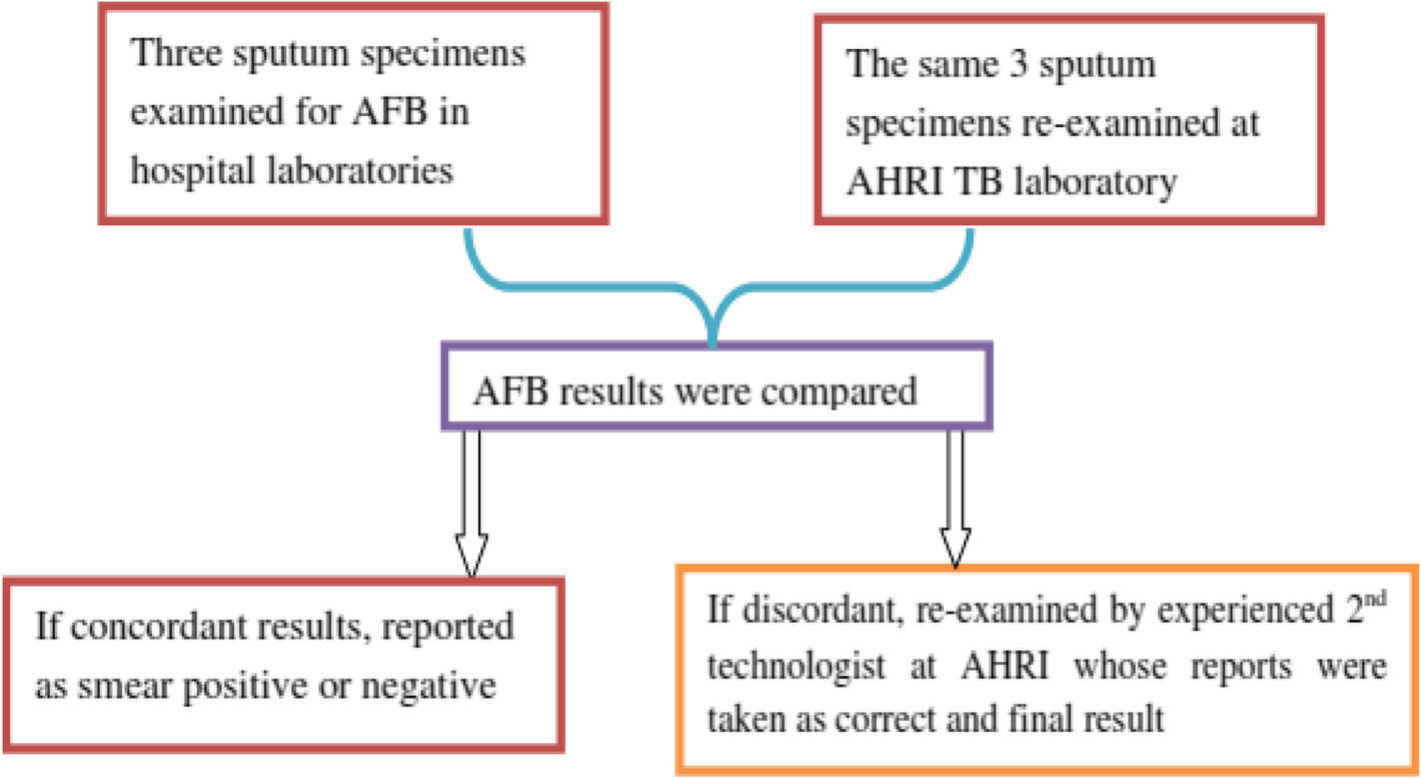
AFB examination algorithm at hospital and research laboratories

Three sputum specimens from each patient were collected; morning sputum at home, and two spot specimens spaced by 30 min after the patient delivered the morning specimen. A pair of smears was prepared from each specimen, air dried and heat fixed. One slide of each pair was examined at hospital laboratories using Ziehl Neelsen (ZN) staining technique. The rest three smears were transported and examined blindly at Armauer Hansen Research Institute (AHRI) TB laboratory in Addis Ababa, Ethiopia (Fig. 1). The results were interpreted as negative (no AFB), scanty (1–9 AFB/100 field), 1+ (10–99 AFB/100 field), 2+ (1–10 AFB/field), 3 + (> 10 AFB/field) [4].

### Chest radiography

All patients were set for Chest X-ray examinations to identify lung cavitation, measure cavity size and count the number of cavities. A senior radiologist at Kharamara hospital examined all the X-ray films and digital images. The radiologist was blinded to radiologic and AFB results reported during the standard initial diagnosis. Sample of X-ray films (*n* = 41) were randomly picked and blindly re-checked by another radiologist to ensure the reliability of X-ray readings. As of rechecking, we found levels of 95.1% [84.6–100%] kappa agreement for cavity identification, 0.84 [0.64, 0.93] Cohen’s kappa coefficient for cavity size, and 85.7% [63.7–96.9%] Cohen’s proportion of zero difference for cavity count.

### Measurements

#### Pulmonary cavitation

Is presence of an air-containing lucent space within a consolidation or a mass or nodule surrounded by infiltrate or fibrotic wall identified upon radiological examination [18, 24]

#### Smear positivity

Was confirmed if at least one sputum smear was positive for AFB upon rechecking at hospital and TB research laboratories [4]

#### Diagnosis delay

Is the time duration from onset of pulmonary symptoms to date of diagnosis. Patients were asked the first dates of illness onset, healthcare provider consultation, and diagnosis. If patients were unsure of the dates, they were assisted to associate with main religious or cultural events. Patient cards were also cross-checked. Then the interval between illness onset and diagnosis was calculated. Cough was the benchmark of illness onset, but another symptom that compelled the patients to seek care was also used in the absence of cough.

#### Mid-upper arm circumference (MUAC)

Was measured using inelastic paper tapes and categorized at 23 cm cutoff (low if MUAC ≤23) according to FANTA’s recommended cutoff to assess undernutrition in adults [25]

#### Body mass index (BMI)

Was computed from weight (kilograms) and height (meter-square) measures, and categorized at 18.5 Kg/M^2^ cutoff (low if BMI < 18.5) according to FANTA’s recommended Cutoff to assess undernutrition in Adults [25].

### Data processing and analysis

Data were double entered and validated using EpiData version 3.1; and analyzed using Stata version 14 (*StataCorp, College Station, Texas 77,845 USA*). Descriptive statistics was done to summarize delays in diagnosis, cavitation, smear positivity and explanatory variables. Since cavitation and smear positivity were common outcomes (> 10%), prevalence ratios with 95% confidence intervals (CI) were used to reveal the association of delay and other factors associated with cavitation and smear positivity. Two log-binomial regression models were fitted using cavitation and smear positivity as separate outcomes of interest. Significance was determined at *p* ≤ 0.05, and variables with *p* ≤ 0.2 in bivariate analyses were included in final multivariable models. Receiver Operating Characteristic (ROC) curve was used to determine the discriminative ability of delay to predict patients with cavitation and smear positivity. Threshold delays were determined at given sensitivity (> 70%) and maximum positive likelihood ratio.

## Results

### Patient characteristics

Totally, 434 pulmonary TB patients were included; all and 421 of them had complete chest radiography and AFB results, respectively. The median age of participants was 30 years, ranged 15 to 82 years. The majority (62.9%1) was male, and close to half (46.5%) were reliant on pastoralism (of which, 36% nomadic). Regarding clinical characteristics, majorities were new cases (90.3%), and 2.3% were co-infected with HIV (Table 1).

**Table 1:** Socio-demographic and clinical characteristics of TB patients in Somali region, Ethiopia, December 2017 to October 2018

Characteristics of patients (*N* = 434)	Frequency (%)
Sex	
Male	273 (62.9)
Female	161 (37.1)
Age group	
15 to 23	115 (26.5)
24 to 30	112 (25.8)
31 to 50	123 (28.3)
51+	84 (19.4)
Literacy level	
Illiterate	267 (61.5)
Primary	45 (10.4)
Secondary	64 (14.7)
Tertiary	58 (13.4)
Marital status	
Single	131(30.2)
Married	265 (61.1)
Divorced/separated/widowed	38 (8.7)
Residence	
Rural	215 (49.5)
Urban	215 (49.5)
Refugee/displaced	4 (1.0)
Livelihood	
Pastoralism	202 (46.5)
Other	232 (53.5)
Income	
Saving	54(12.5)
Income = expense	303 (69.8)
Indebt	77 (17.7)
Cough	
Yes	412 (94.9)
No	22 (5.1)
Haemoptysis	
Yes	33 (7.6)
No	401 (92.4)
Chest pain	
Yes	250 (57.6)
No	184 (42.4)
Breathing difficulty	
Yes	93 (21.4)
No	341 (78.6)
Functional status	
Good	60 (13.8)
Ambulatory	360 (83.0)
Bedridden	14 (3.2)
Treatment category	
New	392 (90.3)
Retreatment	42 (9.7)
History of tuberculosis	
Yes	65 (15.0)
No	369 (85.0)
HIV status	
Positive	10 (2.3)
Negative	422 (97.2)
Unknown	2 (0.5)
Diabetes mellitus	
Yes	16 (3.7)
No	412 (94.9)
Unknown	6 (1.4)
Smoking history	
Ever smoker	45 (10.4)
Never smokers	389 (89.6)
Khat chewing	
Ever chewer	58 (13.4)
Never chewer	376 (86.6)

The median diagnosis delay from onset of illness to the date of TB diagnosis was 49 days (IQR = 33–70), ranged 8 to 362 days. Four patients had diagnosis delay greater than 254 days.

### Patient infectiousness (cavitation and smear positivity)

Of the 434 PTB cases, 45.6% [95%CI: 40.9–50.4%] had single-to-five cavities (mean, 1.8 ± 0.9 cavities) with mean diameter of 2.8 ± 1.0 cm. Of the non-cavitary cases, 5.5% had consolidated lesions but not duly branded as cavity. Whereas, 42.0% [95%CI: 37.3–46.9%] of patients were smear positive upon rechecking. In hospital laboratories, 19.2% of smear positive patients were misidentified as smear negative and 2.9% of smear negative as smear positive. Smear positivity was multifold among cavitary patients (75.3%) compared with non-cavitary counterparts (13.7%), [*P* < 0.001]. Conversely, cavitation was 82.5% [95%CI: 76.1–87.8%] among smear positive patients. AFB examination correctly identified 75.3% [95%CI: 68.6 ˗ 81.2%] of patients with cavitation (sensitivity) and 86.3% [95%CI: 81.2–90.5%] without cavitation (specificity) (Table 2).

**Table 2:** Smear positivity versus cavitation results matrix

	Cavitary TB		Total
	Yes (%)	No (%)	
AFB result			
Positive	146 (75.3)	31 (13.7)	**177 (42.0)**
Negative	48 (24.7)	196 (86.3)	**244 (58.0)**
Total	**194**	**227**	**421**

Key: The percentages indicate the proportions of smear positive and negative patients among Cavitary and non-Cavitary cases

### Delay and other factors associated with patient infectiousness

Cavitation was significantly associated with diagnosis delay [*p* < 0.001]. When categorized at 25th, 50th and 75th percentiles, cavitation was continually increased among patients with diagnosis delay of 31–49 days [APR (95%CI) =1.8 (1.2–2.8)], 50–70 days [APR (95%CI) =2.4 (1.6–3.7)] and 71+ days [APR (95%CI) = 2.7 (1.8–4.1)], given diagnosis delay ≤30 days as reference. Ninety percent (90%) of cavitary patients had delay above 30 days (Table 3). Smear positivity was also significantly increased among patients with diagnosis delay above 49 days [APR (95%CI) = 1.3 (1.1–1.6), *p* = 0.02] (Table 4).

**Table 3:** Factors Associated with Pulmonary cavitation of TB patients in Somali region, Ethiopia, December 2017 to October 2018

Characteristics (n = 434)	Total PTB cases n (%)	Cavitary TB n (%)	*P-*value	PR (95% CI)	*P-*value	APR (95% CI)
Sex						
Female	161 (37.1)	70 (43.5)	0.49*	1	–	–
Male	273 (62.9)	128 (46.9)		1.1 (0.9, 1.3)		
Age						
15 to 35	251 (57.8)	125 (49.8)	0.04	1.3 (1.01, 1.6)	0.04	1.3 (1.01, 1.6)
36+	183 (42.2)	73 (39.9)		1		1
Livelihood						
Pastoralism	202 (46.5)	96 (47.5)	0.45*	1.1 (0.9, 1.3)	–	–
Non-pastoralism	232 (53.5)	102 (44.0)		1		
Smoking						
Ever smoker	45 (10.4)	23 (51.1)	0.40*	1.1 (0.8, 1.5)	–	–
Never smoker	389 (89.6)	175 (45.0)		1		
BCG scar						
Yes	52 (12.0)	26 (50.0)	0.48*	1.1 (0.8, 1.5)	–	–
No	382 (88.0)	172 (45.0)		1		
Chronic diseases (HTP/CHD/CRD)						
Yes	20 (4.6)	12 (60)	0.13	1.3 (0.9, 1.9)	0.006	1.8 (1.2, 2.6)
No	414 (95.4)	186 (44.9)		1		1
MUAC _female_ (*n* = 161)						
Low (≤23 cm)	93 (57.8)	51 (54.8)	0.002	2.0 (1.3, 3.0)	0.01	1.8 (1.13, 2.8)
High (> 23 cm)	68 (42.2)	19 (27.9)		1		1
MUAC _male_ (*n* = 273)						
Low (≤23 cm)	154 (56.4)	74 (48.1)	0.63*	1.1 (0.8, 1.4)	–	–
High (> 23 cm)	119 (43.6)	54 (45.4)		1		
BMI						
Low (< 18.5)	304 (70.0)	149 (49.0)	0.04	1.3 (1.01, 1.7)	0.23	1.2 (0.9, 1.5)
High (≥18.5)	130 (30.0)	49 (37.7)		1		1
Prior history of TB						
Yes	65 (15.0)	31 (47.7)	0.71*	1.1 (0.8, 1.4)	–	–
No	369 (85.0)	167 (45.3)		1		
Delay in diagnosis (days)						
30 or less	90 (20.7)	20 (22.2)	1	1	–	1
31 to 49	138 (31.8)	58 (42.0)	0.004	1.9 (1.2, 2.9)	0.006	1.8 (1.2, 2.8)
50 to 70	98 (22.6)	54 (55.1)	< 0.001	2.5 (1.6, 3.8)	< 0.001	2.4 (1.6, 3.7)
71 or more	108 (24.9)	66 (61.1)	< 0.001	2.8 (1.8, 4.2)	< 0.001	2.7 (1.8, 4.1)

Key: MUAC and BMI Cutoffs were 23 cm and 18.5 Kg/M2; Delay categorized at 25th, 50th and 75th quartile days;1 indicates reference category; *indicates the variable not included in multivariable regression analysis; *BCG* Bacillus Calmette-Guerin, *PR* Prevalence ratio, *APR* Adjusted prevalence ratio, *HTP/CHD/CRD* Hypertension/Chronic Heart Disease/Chronic Renal Disease

**Table 4:** Factors Associated with Sputum Smear positivity of TB patients in Somali region, Ethiopia, December 2017 to October 2018

Characteristics (n = 434)	Total PTB cases n (%)	Smear positive TB n (%)	*P-*value	PR (95% CI)	*P-*value	APR (95% CI)
Sex						
Female	157 (37.3)	58 (36.9)	0.11	1	0.17	1
Male	264 (62.7)	119 (45.1)		1.2 (0.9, 1.5)		1.2 (0.9, 1.5)
Age						
15 to 35	245 (58.2)	119 (48.6)	0.002	1.5 (1.2, 1.9)	0.007	1.4 (1.1, 1.8)
36+	176 (41.8)	58 (33.0)		1		1
Livelihood						
Pastoralism	194 (46.1)	81 (41.8)	0.91*	0.98 (0.79, 1.24)	–	–
Non-pastoralism	227 (53.9)	96 (42.3)		1		
Smoking						
Ever smoker	43 (10.2)	22 (51.2)	0.17	1.2 (0.9, 1.7)	0.28	1.2 (0.8, 1.6)
Never smoker	378 (89.8)	155 (41.0)		1		1
BCG scar						
Yes	52 (12.4)	23 (44.2)	0.61*	1.01 (0.7, 1.4)	–	–
No	369 (87.6)	154 (41.7)		1		
Chronic diseases (HTP/CHD/CRD)						
Yes	20 (4.8)	8 (40.0)	0.85*	0.95 (0.5, 1.6)	–	–
No	401 (95.2)	169 (42.1)		1		
MUAC						
Low (≤23 cm)	235 (55.8)	119 (50.6)	< 0.001	1.6 (1.3, 2.1)	0.003	1.5 (1.2, 1.9)
High (> 23 cm)	186 (44.2)	58 (31.2)		1		1
BMI						
Low (< 18.5)	294 (69.8)	135 (45.9)	0.02	1.4 (1.1, 1.8)	0.04	1.3 (1.01, 1.7)
High (≥18.5)	127 (30.2)	42 (33.1)		1		1
Prior history of TB						
Yes	65 (15.4)	22 (33.8)	0.17	0.8 (0.5, 1.1)	0.25	0.8 (0.6, 1.2)
No	356 (84.6)	155 (43.5)		1		1
Delay in diagnosis (days)						
49 or less	222 (52.7)	83 (37.4)	0.04	1	0.02	1
50 or more	199 (47.3)	94 (47.2)		1.3 (1.01, 1.6)		1.3 (1.1, 1.6)

Key: MUAC and BMI Cutoffs were 23 cm and 18.5 Kg/M^2^; Delay categorized at median day;1 indicates reference category; *indicates the variable not included in multivariable regression analysis; *BCG* Bacillus Calmette-Guerin, *AFB* Acid-Fast Bacilli, *PR* Prevalence ratio, *APR* Adjusted prevalence ratio, *HTP/CHD/CRD* Hypertension/Chronic Heart Disease/Chronic Renal Disease

Other factors associated with cavitation were age ≤ 35 years [APR (95%CI) = 1.3(1.01–1.6)], chronic diseases *(Hypertension/chronic Heart/Renal Disease)* [APR (95%CI) =1.8 (1.2–2.6)], and low MUAC (*in female only*) [APR (95%CI) =1.8 (1.2–2.8)] (Table 3). Similarly, smear positivity was associated with age ≤ 35 years [APR (95%CI) =1.4 (1.1–1.8)], low BMI [APR (95%CI) =1.3 (1.01–1.7)], and low MUAC [APR (95%CI) =1.5(1.2–1.9)] (Table 4).

### Discriminative ability of delay to predict cavitation and smear positivity

**Fig. 2:**
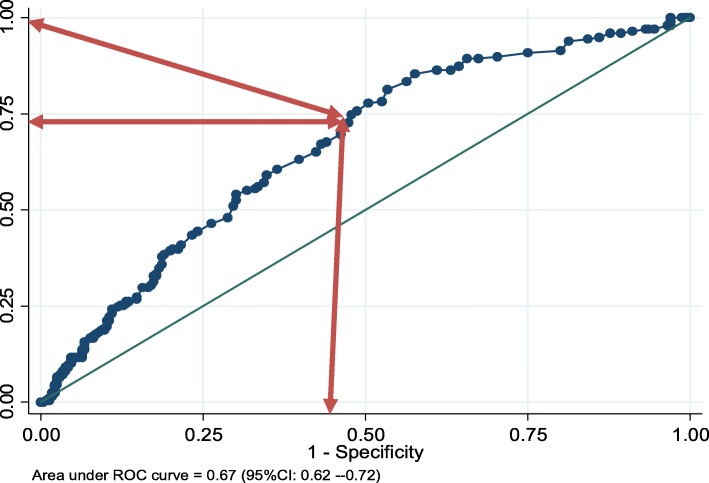
ROC curve (horizontal = l-specificity, vertical = sensitivity) illustrating diagnosis delay as predictor of pulmonary cavitation

**Fig. 3:**
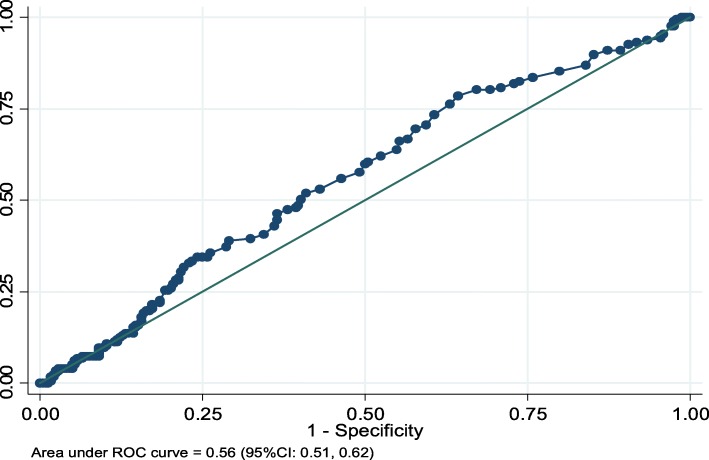
ROC curve (horizontal = l-specificity, vertical = sensitivity) illustrating diagnosis delay as predictor of smear positivity

The ROC curve shows that diagnosis delay discriminated pulmonary cavitation, and the area under the ROC curve (AUC) was 0.67 [95%CI: 0.62–0.72]. The threshold delay was determined at 43 days, at which the resulting sensitivity, specificity and the likelihood ratio for positive test result were 74.6, 52.1% and 1.6, respectively (Fig. 2). At this cutoff point, delay can correctly classify 62.4% of patients with or without cavitation, and 60.0% of patients delayed longer than 43 days without obtaining diagnosis. Nonetheless, diagnosis delay revealed poor discriminative ability to predict smear positivity [AUC (95%CI): 0.56 (0.51–0.62)] (Fig. 3).

## Discussion

The present finding reveals that close to half (45.6%) of all and 82.5% of smear positive patients with pulmonary TB had one or more cavities; 42% were smear positive, and half of patients delayed more than 7 weeks (49 days) and few nearly a year without medical care. Cavitation was increased in patients who delayed longer than 30 days and optimized at threshold delay of 43 days. Similarly, smear positivity was increased in patients who delayed above 7 weeks.

Cavitation and smear positivity were reciprocally illustrative, and about half of patients had intricate form of the disease and were capable of transmitting TB. The cavitation rate matches with the maximum assumption of 50% rate that happens if patients do not receive treatment during the entire course of the disease [26, 27]. It surpassed 34.0% [28] and 21% [29] findings reported elsewhere. Cavitation among smear positive patients was almost twice to previous reports of 49.9% [29] and 38.3% [30] in other places. Nonetheless, smear positivity was comparable to other reports in Ethiopia [31–33]. This implies that many patients in the study area received medical care after the disease gets severe and hazardous.

Our study reveals that the elevated levels of cavitation and smear positivity were attributed to delays in diagnosis and treatment. The threshold delay (43 days) that optimizes cavitation was below the median delay (49 days), or 60% of patients had diagnosis delay longer than the threshold delay. Extreme delay does not only increase infectiousness as figured out here or elsewhere [12, 13], but it also prolongs the contagious period and contact time between patients and contacts [16]. The majority of infectious patients (90% of cavitary and 84% of smear positive) delayed longer than a month with average six-plus household members. Household transmission could be, therefore, higher in pastoral communities bearing in mind their petite transitory huts with closed indoor spaces. Moreover, the high delays could threaten treatment outcomes. The high cavitation due to delay could result in treatment failure/relapse, emergence of drug resistance and permanent lung impairments [28]. Recent findings call for extended treatment of cavitary TB with a combination of new drugs and strategies [34], yet no special strategy is currently in place in Ethiopia. Hence, the effects of delay on treatment outcomes and household transmission should be evaluated in such areas.

In addition to delay, cavitation and smear positivity were also associated with younger (≤35 years), under-nutrition and the presence of chronic diseases co-morbidity after adjusting for potential confounders. The risk of cavitation is likely higher in younger and immune competent patients [35, 36]. This is because concomitant and weakening physical conditions blunt inflammatory responses in older people [37]. The association of cavitation and smear positivity with undernutrition could be either way; undernutrition led to immune-deficiency and enhanced disease progression [38, 39], or the disease itself led to under-nutrition [40]. Unlike this study, other studies revealed diabetes, smoking and low income [41–43] as factors associated with cavitation and smear positivity. The reason why these were not witnessed in the current study might be due to the small number of cases that cohabit these factors.

However, this study is subjected to certain limitation. The reduced sensitivity of Chest X-ray, and salivary sputum and missed smears might underestimate cavitation, and smear positivity, respectively. Recall bias might influence the precision of delay. Moreover, the proportion of pastoralists seems less represented due to the reduced case notification that was observed during dry seasons when they move for pasture and water.

## Conclusion

This study highlights delay in diagnosis and treatment remained high and substantially increased infectiousness of patients with pulmonary tuberculosis in pastoral settings in Ethiopia. Delay of 43 days looks the threshold delay at which risk of cavitation increases significantly, but around two-third of patients obtained diagnosis after this cutoff point. The high levels of cavitation and smear positivity because of excessive delays suggests a threat of high disease transmission and adverse treatment outcomes in the pastoral area. Thus, delay in diagnosis and treatment of TB cases should be targeted for effective control of the disease and prevent the risk of transmission in the pastoralist areas of the country. Strategies that suit socio-cultural needs of pastoralists such as mobile screening services need to be adapted to ensure early detection and treatment of TB.

## Supplementary information

**Additional file 1.** Questionnaire

## Data Availability

The dataset supporting the conclusions of this article is included within the article. The collected data contain confidential information, and consent has not been obtained for public sharing of raw data with identifiers. However, the datasets used and/or analyzed are available at the hands of the corresponding author and can be shared upon reasonable requests.

## Abbreviations

AFB: Acid Fast Bacilli
AHRI: Armauer Hansen Research Institute
APR: Adjusted Prevalence Ratio
AUC: Area Under the Curve
BCG: Bacillus Calmette-Guerin
BMI: Body Mass Index
CI: Confidence Interval
CM: Centimeters
DOTS: Directly Observed Therapy-Short Course
FMOH: Federal Ministry of Health
HIV: Human Immunodeficiency Virus
IQR: Inter-Quartile Range
Kg: Kilogram
M: Meter
MDR: Multi-Drug Resistant
MTB: *Mycobacterium tuberculosis*
MUAC: Mid-Upper Arm Circumference
NTP: National TB Control Program
PR: Prevalence Ratio
PTB: Pulmonary Tuberculosis
ROC: Receiver operating characteristic
SRS: Somali Regional State
TB: Tuberculosis
WHO: World Health Organization
