# Supplementary material for: Delay in diagnosis of pulmonary tuberculosis increases the risk of pulmonary cavitation in pastoralist setting of Ethiopia
Source: BMC Pulm Med. 2019 Nov 6;19:201. doi: 10.1186/s12890-019-0971-y (PMC6836413; doi:10.1186/s12890-019-0971-y)
Supplement: Supplementary file 1 — Additional file 1. Questionnaire [file 12890_2019_971_MOESM1_ESM.docx]

# Questionnaire

The questionnaire contains questions for patient interviews, record reviews, anthropometry recording forms, and report forms for chest X-ray and AFB examinations.

1. Patient ID (TB register no): _____________________
2. Code of the health facility: _____________________
3. Name of interviewer: _____________________­­­­­­­­­­­­­­_____
4. Date of the interview (dd/mm/yy): _____________________
5. Does the patient have any other pulmonary disease such as pneumonia and lung cyst? If yes, discontinue interview [*look patient medical record*]

**Socio-demographic Characteristics**

| **S. No** | **Question** | | **Responses** | **Remark** |
| --- | --- | --- | --- | --- |
|  | Age of the patient *[15 years and above]* | | ______________ (full years) |  |
|  | Sex of the index case | | 1. Female 2. Male |  |
|  | How many household members are living with you? | | ______________(in numbers) |  |
|  | What is your educational level? | | 1. Do not read and write 2. Read and write 3. Primary (grade 1 to 6) 4. Secondary (grade 7 to 10) 5. Tertiary (10 +) |  |
|  | Marital status | | 1. Married 2. Single 3. Divorced/separated 4. Widowed |  |
|  | Where do you live? | | 1. Rural 2. Suburban 3. Urban 4. Homeless/displaced 5. Refugee |  |
|  | What is your occupation? | | 1. Pastoralist 2. Technical/professional 3. Laborer 4. Unemployed 5. Student | If the answer is other than 1, go to Q # 109 |
|  | If 1 for Q#107, type of pastoralist | | 1. Nomadic 2. Agro-pastoral |  |
|  | How much is your household income? | | 1. Savings 2. Income = expenses 3. In debt |  |
|  | What is the type of the nearest health facility? | | 1. Health post 2. Health center 3. Clinic 4. Hospital |  |
|  | How long does it take to reach the nearest public health facility from your home (*one way walking time*) | | _____________ (record in minutes) |  |
|  | Does the nearest health facility (answered at Q#110) have TB diagnosis and treatment services? | | 1. Yes 2. No 3. I don’t know |  |
| **History of current illness: Chief symptoms and date of onset of the current illness (Circle the answer and record the date of symptom onset if the answer is ‘Yes’ for the particular symptom).** | | | | |
|  | Which of the following sign and symptoms occurred during the onset of your current illness? (*Multiple answers possible)* | | |  |
|  | 113a. Cough | 1. Yes 2. No  If yes, (dd/mm/yy)_____________ | |  |
|  | 113b. Haemoptysis /coughing blood | 1. Yes 2. No  If yes, (dd/mm/yy)____________ | |  |
|  | 113c. Chest pain | 1. Yes 2. No  If yes, (dd/mm/yy)____________ | |  |
|  | 113d. Breathing difficulty | 1. Yes 2. No  If yes, (dd/mm/yy)_____________ | |  |
|  | 113e. Weight loss | 1. Yes 2. No  If yes, (dd/mm/yy)____________ | |  |
|  | 113f. Night sweating | 1. Yes 2. No  If yes, (dd/mm/yy)____________ | |  |
|  | 113g. Fatigue | 1. Yes 2. No  If yes, (dd/mm/yy)____________ | |  |
|  | 113h. Fever | 1. Yes 2. No  If yes, (dd/mm/yy)____________ | |  |
|  | 113i. Loss of appetite | 1. Yes 2. No  If yes, (dd/mm/yy)____________ | |  |
|  | Which of the above symptom(s) made you seek initial healthcare mainly? (*use* *the* *above code* e.g. 113a, 113b…) | _____________________ | |  |
|  | What was the date you first visited a formal HCP [*hospital, health center, private clinics with TB diagnostic services*] for the symptom(s) for the symptom(s)? | dd/mm/yy: ___________________ | |  |
|  | What was the date when your illness was diagnosed as TB?*[can be found on the patient card or record book]* | dd/mm/yy: ___________________ | | The date the patient was diagnosed as TB patient |

**Computing time delays:**

The interviewer will compute the time delays in diagnosis. The delays should be computed in number of days. If cough is mentioned as initial symptom, use it to compute time delays but if not, the date of the main symptom that compelled the patient to seek medical consultation should be used to calculate time delays.

1. Patient delay (days from Q#113a if cough/any other to Q#115): ______________ (days)
2. Health system delay (days from Q#115 – Q#116): _____________ (days)
3. Total diagnosis delay (days from Q#113a if cough/any other to Q#116): _______ (days)

| **S. No** | **Question** | | | | **Response** | | | **Remark** | |  |
| --- | --- | --- | --- | --- | --- | --- | --- | --- | --- | --- |
| **Substance use** | | | | | | | | | |  |
|  | Do you smoke cigarette/shisha? | | | | 1. Never 2. Quitted smoking 3. Current smoker | | | If 1& 2, go to Q#123 | |  |
|  | If current smoker, specify amount of daily consumption (*average number of cigarettes/day*) | | | | ___________________ | | |  | |  |
|  | Duration of smoking | | | | Years-----------; months------------ | | |  | |  |
|  | Do you chaw Khat? | | | | 1. Never 2. Quitted chewing 3. Current chewer | | | If 1 & 2, go to Q#126 | |  |
|  | If chewer, frequency of chewing | | | | 1. Daily 2. At least once a week 3. Occasionally | | |  | |  |
|  | Duration of chewing | | | | Years-----------; months------------ | | |  | |  |
|  | Do you drink alcohol? | | | | 1. Never 2. Quitted drinking 3. Current drinker | | | If 1 & 2, go to Q# 130 | |  |
|  | If drinker, what type of alcohol do you usually drink? | | | | 1. Local (*Araki, Teji, Tela…*) 2. Beer 3. Other (specify)_________ | | |  | |  |
|  | If drinker, frequency of drinking | | | | 1. Daily 2. At least once a week 3. Occasionally | | |  | |  |
|  | Duration of alcohol drinking | | | | Years-----------; months------------ | | |  | |  |
| **Health care seeking practice (behavior) following onset of TB symptoms** | | | | | | | | | | |
|  | What did you do first when you developed the initial symptoms for your current illness?  (*First action before initial diagnosis*) | | | 1. Visit HCP 2. Visit Health extension worker 3. Self-medication 4. Take traditional/home remedy 5. Visit drug vendor (pharmacy) 6. Private clinic with no TB diagnosis 7. Other (specify) ___________ | | | *HCP is a health care provider who works at hospital, health center, and Private hospital/ clinic with TB diagnosis service*  If 1, go to Q.134 | | | |
|  | If not HCP (codes 2,3,4,5, 6, 7), reasons for initial non-consultation of HCP following the onset of symptoms? | | | 1. Lack of confidence in getting cured 2. Too far 3. Too busy/long waiting time 4. Costly service 5. Bad experience 6. Other (specify)------------------ | | |  | | | |
|  | Who told you to do it first (*mentioned in Q#132*)? | | | 1. Nobody (myself) 2. Family member 3. Neighbor/friend 4. HEW 5. Traditional/religious healer 6. Drug vendor 7. Other (specify)----------------- | | |  | | | |
|  | Have you sought care from traditional healer or religious leader for the current illness prior to HCP consultation? | | | 1. Yes 2. No 3. I don’t remember | | |  | | | |
|  | What is the health facility of the healthcare provider that you obtained the first consultation? | | | 1. Health center 2. Hospital 3. Private hospital/clinic with TB services | | |  | | | |
|  | What is the health facility of the Health Care Provider who made the initial TB diagnosis | | | 1. Health center 2. Hospital 3. Private facility with TB service | | |  | | | |
|  | Action made by Health Care Provider who made the initial TB diagnosis | | | 1. Sputum examination 2. X-ray 3. Both 4. Referral 5. Others (specify)----------------- | | |  | | | |
| **Clinical presentation** | | | | | | | | | | |
|  | What symptoms do you have now?  *[More than one answer possible]*  (*check patient history card*) | | | 1. Cough 2. Breathing difficulty 3. Chest pain 4. Bloody sputum (haemoptysis) 5. Excessive sweating at night 6. Fatigue 7. Weight loss 8. Fever 9. Other (specify)--------------- | | |  | | | |
|  | If cough, how long has been the cough duration? | | | ------------------------ days | | |  | | | |
|  | How do you rate the severity of your illness? | | | 1. None 2. Mild 3. Moderate 4. Severe | | |  | | | |
|  | What is the physical condition (functional status) of the patient? | | | 1. Good 2. Ambulatory 3. Bedridden | | |  | | | |
|  | What is the TB treatment category of the patient  (see *patient record*) | | | 1. New 2. Retreatment 3. Transferred in | | |  | | | |
|  | Did you have a family member who had history of TB prior to you | | | 1. Yes 2. No 3. I don’t know | | |  | | | |
|  | Chest X-ray examination result at initial diagnosis (see *patient record*) | | | 1. Normal 2. Indicative of TB 3. Pulmonary cavitation 4. Not done | | |  | | | |
|  | AFB smear result at initial diagnosis (see *patient record*) | | | 1. Negative 2. Positive 3. Unknown | | |  | | | |
| **Patho-Physiologic Conditions:** the data can be obtained from self-report of patients or patient record. | | | | | | | | | | |
|  | In addition to TB, do you have a co-morbidity of other pulmonary diseases?  *(It can also be found on the patient medical record)* | | | 1. None 2. Pneumonia 3. Asthma 4. Bronchitis 5. Lung cyst 6. Other (specify)---------------- | | | If 1, go to Q #147 | | | |
|  | If any of the above co-morbidities, when did it start? | | | 1. Before TB 2. After TB 3. I do not remember | | |  | | | |
|  | Do you have diabetes mellitus?  *(can be found on the patient record or check by rapid test)* | | | 1. Yes 2. No 3. Unknown | | |  | | | |
|  | Do you have a diagnosed malignancy? | | | 1. Yes 2. No | | |  | | | |
|  | Hypertension *(can be measured or from Patient record)* | | | 1. Yes 2. No 3. Unknown | | |  | | | |
|  | Do you have any chronic diseases? | | | 1. None 2. Chronic heart disease 3. Chronic renal disease 4. Liver disease 5. Other (specify)---------- | | |  | | | |
| **Immune Status** | | | | | | | | | | |
|  | BCG scar | | | 1. Yes 2. No | | | *Observe the scar on the upper right arm* | | | |
|  | HIV status (*take from patient record but do not ask the patient*) | | | 1. Positive 2. Negative 3. Unknown | | |  | | | |
|  | Have you taken Immune-suppressive therapy [*look patient record*] | | | 1. Yes 2. No 3. Unknown | | |  | | | |
|  | Had you ever developed TB sometime before? | | | 1. Yes 2. No 3. I do not remember | | |  | | | |
| **Nutritional Status** | | | | | | | | | | |
|  | MUAC | | | ------------------- centimeters | | |  | | |  |
|  | Body Mass Index (BMI) | | | a. Weight -------- (KG)  b. Height --------- (M)  c. BMI----------------(KG/M2) | | | BMI = W (KG)  H^2^ (M) | | |  |
| **Household conditions** | | | | | | | | | |  |
|  | House type | | | 1. Traditional hut 2. Wood plus mud with metal roof 3. Cement/concrete | | | If 1, go to Q # 334 | | |  |
|  | How many total rooms does the household have? | | | ------------------- (no. of rooms) | | |  | | |  |
|  | How many rooms in the household are used for sleeping? | | | ------------------- (no. of rooms) | | |  | | |  |
|  | Does the sleeping room (s) have windows? | | | 1. Yes 2. No | | |  | | |  |
|  | Do you share a sleeping room with other family members? | | | 1. Yes 2. No | | |  | | |  |
|  | The presence of cough | | | 1. Yes 2. No | | |  | | |  |
|  | Where do you expel your sputum? | | | 1. Anywhere as it comes 2. Inside prepared container 3. Other(specify)------------ | | |  | | |  |
|  | Do you think that you could potentially transmit TB to your family? | | | 1. Yes 2. No 3. I do not know | | | If 2 & 3, go to Q # 166 | | |  |
|  | If yes, how could it be transmitted to others?  *(Don’t read choices and multiple answer possible)* | | | 1. Airborne droplets released infected person 2. Contaminated food/ drink 3. Sexual intercourse 4. Other (specify)---------- 5. I do not know | | |  | | |  |
|  | What do you do to prevent transmission to your family members or others | | | 1. Cover mouth while coughing/ sneezing 2. Separate sleeping 3. Separate meal utensils 4. No thing I do | | |  | | |  |

**Chest X-ray Examination report**

Name of Hospital:______________________________________________

Name of patient:_______________________________________________

Patient ID (TB register no): ______________________________________

| **Chest X-ray examination** | | | |
| --- | --- | --- | --- |
|  | Pulmonary Cavitation | - 1. Yes   2. Undetermined   3. No | If yes, fill the following findings |
|  | If cavity, the quantity of cavities | -------------------------- cavities |  |
|  | If cavity, the diameter of the largest cavity | ----------------------- (centimeters) |  |
| **Radiologist report:** | | | |

Name & signature of Radiologist: ______________________Date: _________________

# AFB EXAMINATION REPORTING FORMAT

Name of Hospital:______________________________________________

Name of patient:_______________________________________________

| **Patient ID (TB register no.)** | **Sputum Smear AFB Result at hospital Laboratory** | | | **Smear type/status** |
| --- | --- | --- | --- | --- |
|  | **Morning** | **Spot** | **Spot** |  |
| Patient ID___________ | 1. Negative 2. Scanty 3. + 4. ++ 5. +++ | 1. Negative 2. Scanty 3. + 4. ++ 5. +++ | 1. Negative 2. Scanty 3. + 4. ++ 5. +++ | 1. Negative 2. Positive |

Name & signature of lab tech: _______________________ Date: _________________

# ----------------------------------------------------------------------------------------------------

# AFB EXAMINATION REPORTING FORMAT

Name of Hospital:______________________________________________

Name of patient:_______________________________________________

| **Patient ID (TB register no.)** | **Sputum Smear AFB Result at AHRI TB Laboratory** | | | **Smear type/status** |
| --- | --- | --- | --- | --- |
|  | **Morning** | **Spot** | **Spot** |  |
| Patient ID __________ | 1. Negative 2. Scanty 3. + 4. ++ 5. +++ | 1. Negative 2. Scanty 3. + 4. ++ 5. +++ | 1. Negative 2. Scanty 3. + 4. ++ 5. +++ | 1. Negative 2. Positive |

Name & signature of lab tech: _______________________ Date: _________________
